# Silver Nanoparticle Regulates Salt Tolerance in Wheat Through Changes in ABA Concentration, Ion Homeostasis, and Defense Systems

**DOI:** 10.3390/biom10111506

**Published:** 2020-11-02

**Authors:** Iram Wahid, Sarika Kumari, Rafiq Ahmad, Sofi J. Hussain, Saud Alamri, Manzer H. Siddiqui, M. Iqbal R. Khan

**Affiliations:** 1 Department of Biosciences, Integral University, Lucknow 226026, India; iramicro@gmail.com; 2 Department of Botany, Jamia Hamdard, New Delhi 110062, India; sarikakumari0987@gmail.com; 3 Centre for Nanoscience and Nanotechnology, Jamia Millia Islamia (A Central University), New Delhi 110025, India; ahmadrafiq38@gmail.com; 4 Department of Botany, Government Degree College, Kokernag, Jammu & Kashmir 192202, India; sjavaidjh@gmail.com; 5 Department of Botany and Microbiology, College of Science, King Saud University, Riyadh 11451, Saudi Arabia; saualamri@ksu.edu.sa (S.A.); manzerhs@yahoo.co.in (M.H.S.)

**Keywords:** ABA, ion homeostasis, salt tolerance, silver nanoparticle, wheat

## Abstract

Salinity is major abiotic stress affecting crop yield, productivity and reduces the land-usage area for agricultural practices. The purpose of this study is to analyze the effect of green-synthesized silver nanoparticle (AgNP) on physiological traits of wheat (*Triticum aestivum*) under salinity stress. Using augmented and high-throughput characterization of synthesized AgNPs, this study investigated the proximity of AgNPs-induced coping effects under stressful cues by measuring the germination efficiency, oxidative-biomarkers, enzymatic and non-enzymatic antioxidants, proline and nitrogen metabolism, stomatal dynamics, and ABA content. Taken together, the study shows a promising approach in salt tolerance and suggests that mechanisms of inducing the salt tolerance depend on proline metabolism, ions accumulation, and defense mechanisms. This study ascertains the queries regarding the correlation between nanoparticles use and traditional agriculture methodology; also significantly facilitates to reach the goal of sustainable developments for increasing crop productivity via much safer and greener approachability.

## 1. Introduction

Climate change and rapid increase in population are pragmatically making a serious threat to the world’s agronomic food security [1]. Salt stress has become a great concern in areas of approximately 1125 million hectares around the world; of which 76 million hectares are affected solely by the anthropogenic activities, resulting in the loss of 1.5 million hectares of arable landareas per year due to sodification and salinization [2]. The paramount anthropic pursuits encompassing immoderate irrigation practices, soil topographic perturbation, fertilizers hackneyed, and impoverished drainage system account for this spread-ability of salinity stress [3]. Salt stress has altered physiological responses such as disruption of plasma membrane integrity, excessive reactive oxygen species (ROS) production, reduced photosynthetic efficiency, decrease in aperture size of stomata and insufficient accessibility of antioxidant enzymes [4]. Moreover, accretion of ROS results in oxidative-burst in cellular compartments and affecting their components such as proteins, DNA, and lipids [5].Additionally, the higher accumulation of sodium (Na^+^) and chloride (Cl^−^) ions in plants cause ionic stress and lead to disturbance in uptake, distribution, availability of essential elements, and impairment in selectivity and integrity of cellular membranes [6]. However, the tolerance mechanism of salinity-stressed plants manifests traits such as the exclusion of excessive salt ions, changes in membrane-permeability to regulate the ionic uptake, synthesis and accumulation of compatible metabolites or osmolytes such as proline, promoting ionic homeostasis, and hormonal regulation including abscisic acid (ABA) governs salt tolerance in plants [7,8]. Among all the phytohormones, the ABA-mediated pathway has been studied in context with salinity-induced stresses due to its critical physiological responses and robust mechanism against stressful environmental cues. The positive correlation between ABA biosynthesis and salinity-tolerance traits is due to various strategies opted by plants under stressful extremities such as inducing the seed dormancy, facilitating in reducing the diffusion of Na^+^ ions from the thick caspasrian strips to endodermis, regulation of turgor pressure, and reducing water-loss via partial or complete closure of stomatal aperture [9]. Furthermore, ABA also modulates the stress-responsive gene-expression which specifically encodes proteins such as dehydrins, ROS-detoxifying enzymes, regulatory proteins, and enzymes involved in phospholipids-mediated signaling pathways. The accumulation of ABA during water-deficit conditions initiates a cascade pathway, leading to active efflux of calcium ions (Ca^2+^), and NO^3−^ ions out-flow; which in turn induces potassium ions (K^+^) efflux action and eventually results in stomatal closure, and thereby, conserving the cellular-water [10].

The study about custom-tailored metabolites or metabolic pathways by exploiting the principles of bioengineering also confers coping-ability in plants. However, the recent progression in the domain of bio-nanotechnology emerges out as a promising way for large-scale improvement in crop varieties through potential-infused strategies owing to the coalescence of nanoparticles (NPs) for sustainable and equitable usage of agricultural resources. However, the increase in commercial synthesis of NPs through physical and chemical approaches has imposed environmental stresses due to their toxic metabolites and byproducts released as the reaction-intermediates [11]. Therefore, transmogrifications in the existing methodologies are needed to reduce the ecological risk through the perspective of environment-friendly approaches. In this regard, plants are the best source as they are free from toxic chemicals and provide natural capping agents for NPs synthesis [12].

Effects of nanoparticles have been reported so far in the agricultural discipline; focusing on enhancing seed germination [13], plant growth [14], and photosynthetic rate [15]. Silver nanoparticles (AgNPs) have remarkably ascendant behavior over existing nanoparticles [16] because of their unique physicochemical properties imparting antimicrobial and antioxidant attributes [17]. Furthermore, the non-toxicity and chemical stability at ambient conditions of AgNPs regarded them as the ‘biocompatible precursors’ for inducing the specific traits responsible for the overall development in plants [18]. The present study was made to investigate the conceivable effects of green synthesized AgNP on salinity-induced oxidative stress in wheat (*Triticum aestivum*) through repression of oxidative stress, accumulation of essential ions, and increased activity of enzymes involved in the metabolism of nitrogen, antioxidants, along with modulation of ABA production and stomatal behaviour.

## 2. Materials and Methods

### 2.1. Wheat Leaves Extract Preparation

Silver nanoparticles (AgNPs) were synthesized by using *Triticum aestivum* L. cv. Pusa Kiran (wheat) leaves extract because of its ease of availability and cost-effectiveness. First, the leaf surface was thoroughly washed with the distilled water to remove the debris and other contaminated organic elements, and then the samples were air-dried at room temperature. For the experiment, approximately 20 g of plant leaves were expurgated into small pieces and homogenized with 20 mL tris buffer (50 mM, pH 7.2) using a pestle and mortar. After that, the Whatman filter paper was used to filter the extract by cleaning the suspended fibrous solid particles and then, 4 °C temperature was maintained to keep this extract for further use. The clear filtrate was centrifuged at 10,000 rpm for 15 min and the supernatant was kept in a tube. Later, different concentrations (20, 40, 60, 80, and 95%) of ammonium sulfate were augmented to the supernatant at 4°C. Then, the solution was centrifuged again at 10,000 rpm after incubation at 4 °C for 12 h and the obtained precipitate was mixed in 1 mLof PBS (50 mM, pH 5.0, and 4 °C) to dissolve. After that, the synthesis of AgNPs was cultivated by this purified protein extract. Then, a 0.2 μM filter (Merck Millipore; St. Louis, USA) was employed to filter the supernatant of the extracted sample.

### 2.2. In Vitro Synthesis of Silver Nanoparticles by Wheat

To get the reaction for the synthesis of AgNPs at 40 °C temperature, by adding varying concentrations (30, 40, and 50 μg/mL) of purified protein in 3 mL reaction mixture having 1 mM silver nitrate (AgNO_3_) in 100 mM phosphate buffer at pH 7.2 was added for 40 h. As a control, a separate similar reaction was performed without the purified protein. The collection of AgNPswas determined by centrifugation (15,000 rpm, 30 min) at the end of the synthesis reaction, then the reaction mixture was washed twice withMilli Q water and the unbound purified protein was cleaned by precipitation with 50% *v*/*v* of absolute ethanol and characterized for further use [19]. The solution changes its color from colorless to brown; this color change was a parameter to monitor the Ag^+^to Ag^0^reduction. Then, the UV–visible spectroscopy (Kyoto, Japan) confirmed its formation.

### 2.3. Characterization of Green Synthesized Silver Nanoparticles

Green synthesized AgNPs were characterized by robust techniques such as UV–VIS spectroscopy; transmission electron microscopy, scanning electron microscopy, dynamic light scattering, zeta potential, and Fourier transform infrared spectroscopy.

UV-Vis spectrophotometry was done on a Shimadzu dual-beam spectrophotometer (model UV-1601 PC) and conducted at a resolution of 1 nm. Scanning electron microscopy (SEM) analysis of synthesized AgNPs was operated by the use of a Sigma, Zeiss (Oberkochen, Germany) HR-SEM machine. Further, using transmission electron microscopy (TEM; JEOL JEM-2100, Tokyo, Japan) the size and shape of the synthesized AgNPs were determined, TEM was operated at an accelerating voltage of 200 kV, equipped with a Gatan digital CCD camera (Eindhoven, Netherland). The sample was collected by drying a drop of AgNPs solution on carbon-coated TEM copper grids. A dynamic light scattering (DLS) particle size analyzer (Zeta Sizer Nano-ZS, Model ZEN3600, Malvern Instrument Ltd., Malvern, UK) was the instrument to measure the mean particle size of AgNPs. The sample was acquired in a DTS0112-low volume disposable sizing cuvette of 1.5 mL. Sonic & Material Inc. (New Town, CT, USA) at 30 W for 1 min (10 s on and 5 s off) sonicated these particles. The mean particle size was the average of triplicate measurements for a single sample. The surface charge of AgNPs was measured through a Zeta Sizer Nano-ZS, Model ZEN3600 (Malvern Instrument Ltd., Malvern, UK). Fourier transform infrared (FTIR; Tensor 37, Bruker, Billerica, MA, USA) spectral measurements were carried out to identify the potential biomolecules in wheat which is accountable for reducing and capping AgNPs. A drop of AgNPs solution on a Si substrate was placed to prepare AgNPs film on a Si substrate and evaporation of water was obtained by the procedure of gentle heating and FTIR spectra of the film were documented in the diffuse reflectance mode at a resolution of 4 cm^−1^.

### 2.4. Plant Material and Growth Conditions

Healthy seeds of wheat (*Triticum aestivum* L. cv. Pusa Kiran) were surface sterilized with mercuric chloride solution (0.01%), washed with double-distilled water, and grown in earthen pots (23 cm diameter) filled with reconstituted soil (sand:clay:peat; 70:20:10 by dry weight).The randomized pot experiment was proceeded in the natural environmental conditions with characterized agro-climatic condition of 400 mm annual rainfall in the year of 2019. Plants were treated with 0 mM (control) and 100 mM sodium chloride (NaCl). NaCl at 100 mM was given to plants on alternate days starting from 10 days after sowing (DAS) in the form of modified full-strength Hoagland’s nutrient solution up to 20 days after sowing and the control group of plants was fed with a 300 mL nutrient solution. In the experiment, treatment of AgNP at 300 ppm concentration was applied at 15 DAS on the foliage of both control and NaCl grown plants. At 30 DAS (15 days after AgNP application), morphological and physiological measurements were done with the same age of leaves. The treatments were arranged in complete randomized block design with three replicates for each treatment.

### 2.5. Estimation of Oxidative Stress Markers

The leaf lipid peroxidation was determined via the estimation of thiobarbituric acid reactive substances (TBARS) contents by following the method of [20]. The determination of H_2_O_2_ content was done by following the method of [21]. The details of the methods are given in Supplementary file 1.

The electrolyte leakage was measured by thoroughly washing of leaf samples with sterile water to remove the electrolytes adhered to the leaf surface, followed with weighing and then the samples were immersed in a closed vial containing 10 mL deionized water. After that, the samples were incubated at 25 °C for 6 h using shaker and the initial electrical conductivity (EC) was determined (C_1_) via conductometer. After that, the samples were incubated at 90 °C for 2 h to release all the electrolytes and the final EC was monitored after achieving the equilibrium at 25 °C (C_2_). Then, the statistical value of electrolyte leakage was determined by using the formula, EC= (EC_1_/EC_2_) × 100% [22].

### 2.6. Estimation of Ions Content

The estimate of Na^+^ and Cl^−^ content was determined via the digestion of plant samples using Tri acid mixture (TAM): a sulfuric acid, perchloric acid, and nitric acid mixture in the ratio of 5:4:10. By using a flame photometer, the estimate of Na^+^ content was determined; however, the determination of Cl^−^ content was done by titration against 0.02 N silver nitrate solution using 5% K_2_CrO_4_ as the indicator.

The estimation of K^+^ content was determined by the immediate freezing of the leaf sample in liquid nitrogen. For the determination, the defrost leaf sample was set into 1.5 mL microcentrifuge tubes having basal opening, through which cell sap allows but not the fragments of tissue. The samples were centrifuged at 11,600× *g* for 3 min. in a micro centrifuge. The collected samples were taken for the determination of K^+^ content by using flame photometer. The analysis of K^+^ content was done by following the method of [23].

### 2.7. Estimation of Enzymatic and Non-Enzymatic Antioxidants

About 200 mg leaf samples were crushed in 0.05% (*v*/*v*) Triton X-100 and 1% (*w*/*v*) PVP in potassium-phosphate buffer (100 mM, pH 7.0) containing extraction buffer. The aliquot was spun 15,000× *g* for 20 min at 4 °C in centrifuge and the obtained supernatant was used for the assay of enzymes superoxide dismutase (SOD; EC 1.15. 1.1), ascorbate peroxidase (APX; EC 1.11.1.11), glutathione reductase (GR; EC 1.6.4.2), glutathione peroxides (GPX; EC 1.11.1.9).

The activity of SOD was determined according to the method of [24] and [25]. The activity of APX was determined according to the method of [26]. The determination of GR activity was done by following the method of [27]. The determination of GPX activity was accomplished by the method of [28].

The content of reduced glutathione (GSH) was determined via the enzyme recycling procedure given by Griffith (1980). The content of AsA was estimated via the method of [29] which was adopted from [30].

### 2.8. Estimation of Proline Oxidase, Glutamyl Kinase Activity, and Proline Content

The proline oxidase activity was determined via the method of [31]. The glutamyl kinase activity was determined via the method given by [32]. This method was adopted by [33]. The content of proline was estimated by the method given by [34].

### 2.9. Estimation of Nitrate Reductase, Nitrite Reductase Activities, and Nitrogen Content

The estimation of leaf NR activity was completed by following the method given by [35]. The NiR activity was determined via the method given by [36] which was also adopted by [37].The N content in leaf was estimated in acid-peroxide digested material using the method of [38].

### 2.10. Estimation of Chlorophyll Content and Measurement of Plant Dry Mass

The fully expanded fresh leaf was taken and then cut into strips about 2 mm in width. The extractions of chlorophyll were done from 25–50 mg fresh leaf tissue by dimethyl sulfoxide (DMSO) containing 96% ethanol or 80% acetone. In the non-lethal DMSO method, the leaf tissues were immersed in glass tubes at temperature 65 °C with regular shaking until the leaf tissue became colorless. Record the absorbance spectrophotometrically at 665 and 648 nm. The content of chlorophyll (a and b) was estimated by applying the equation given by [39].

From the pots, the plants were uprooted carefully, and then washed for removing the adhering material and dust; followed by the drying of the sample in hot air oven at 80 °C till constant weight and the weight of dry mass was noted down.

### 2.11. Measurement of Stomatal Traits

The stomatal index (SI) (%) was estimated by using the formula illustrated by [40]:

SI (%) = [S ÷ (E + S)] × 100.(1)

Here, S = number of stomata per unit area; E = number of epidermal cells in the same unit area.

Stomatal frequency (SF) {no. of stomata per mm^2^) was determined by the formula given by [41]:

SF = [S in entire FOV ÷ Area of FOV].(2)

Here, S = Number of stomata per unit area; FOV = field of view = 0.65 mm^2^ (at 40×).

Stomatal shape coefficient (SSC) and the area of stomatal aperture were calculated by the formula given by [42]:

Stomatal Area (SA) = (Stomatal Length (SL) × Stomatal Width (SW) × ∏)/4.(3)

Stomatal Surface Coefficient (SSC) = (SW/SL) × 100.(4)

### 2.12. Estimation of ABA Content

The determination of abscisic acid (ABA) content was done by following the method given by [43].

### 2.13. Estimation of Seed Germination

Germination percentage was estimated by the equation described by [44]:

Germination percentage = (No. of germinated seeds ÷ Total no. seed tested) × 100.(5)

Germination index was calculated by equation given by [45].

Germination index = (germination percentage in each treatment ÷ germination percentage in the control) × 100.(6)

### 2.14. Statistical Analysis

Data were statistically analyzed using analysis of variance (ANOVA) in SPSS statistics software (ver. 17.0) and were presented as treatment mean ± SE (n = 3). The least significant difference (LSD) was calculated for the significant data at *p* < 0.05. The bars showing the same letter are not significantly different by the LSD test at *p* < 0.05.

## 3. Results

### 3.1. Synthesis and Characterization of Nanoparticles

**Figure 1:**
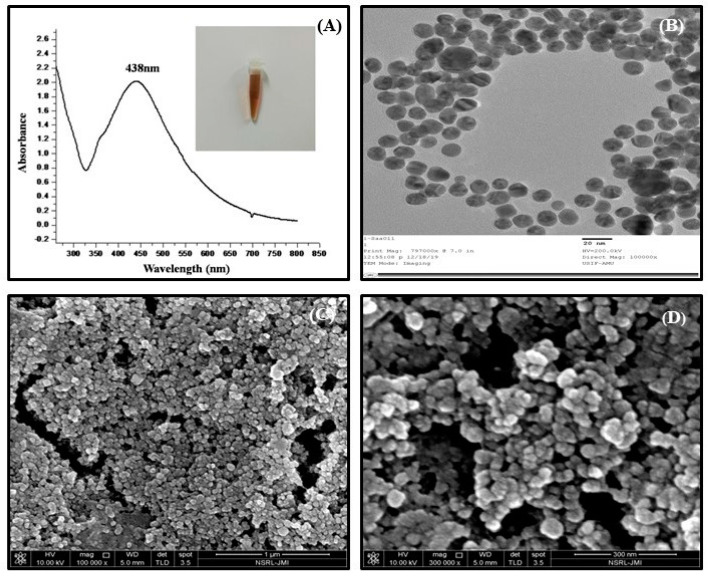
Characterization of AgNPs using (**A**) UV–visible spectrum (λ_max_ = 438 nm), (**B**) transmission electron microscopy (size ~26 nm), (**C**) scanning electron microscopy (size 27–33 nm) at scale bar 1 μm, (**D**) scanning electron microscopy image at scale bar 300 nm.

The incubation of the purified protein with 1 mM AgNO_3_ at 40 °C temperature for 40 h led to the synthesis of silver nanoparticles through monodispersed and high firmness. The synthesis of AgNPs was determined after observing the gradual transformation in color changing from transparent to characteristic brown color in subsequent reaction from incubation with purified protein of *T. aestivum*. The transformation in color was observed due to the surface plasmon resonance activity of AgNPs, which represents the spectroscopic signature of AgNPs formation. Further, it was also confirmed by UV-visible spectroscopy, and surface plasmon resonance absorption band appears at 438 nm (Figure 1A).

The size distribution of synthesized AgNPs (~26 nm) was confirmed by TEM micrographs (Figure 1B). This also assured that AgNPs were spherical in shape and monodispersed and uniformly distributed which was in conformity with the SEM image (Figure 1C). TEM calculates the size of nanoparticles through the direct transmission of electrons that provide the knowledge about the inorganic core too, and does not comprise the hydration layer. In the SEM analysis, the structure of AgNPs as well as their morphological dimensions demonstrated that the average size ranged from 27–33 nm.The shapes of the AgNPs proved to be spherical (Figure 1C,D).

**Figure 2:**
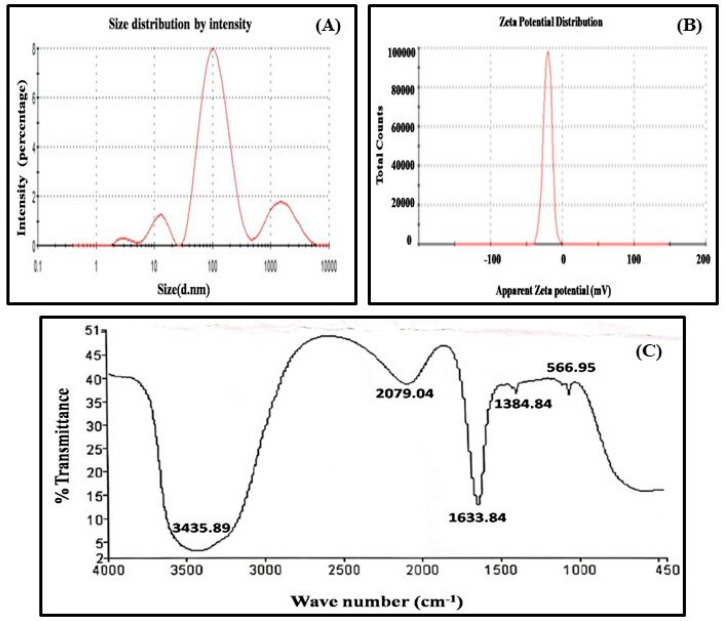
Characterization of AgNPs using (**A**) dynamic light scattering (size 22.25 d.nm), (**B**) zeta potential (−20.3 mv), (**C**) fourier transform infrared spectroscopy.

Moreover, a DLS examination was conducted to get additional information on the particle size distribution and to estimate the hydrodynamic radius of AgNPs that was 22.25 d.nm (Figure 2A). A thin electric dipole layer of the solvent is applied to protect the surfaces of nanoparticles when they are moved through a liquid medium. Therefore, the measurements of nanoparticles achieved under DLS evaluate the hydrodynamic diameter of particles. To get the information easily about the concentration, ionization, exposure, or shielding of charged moieties, distribution, adsorption of the nanoparticles, the Zeta potential instrument was used. The Zeta potential of AgNPs was measured to be −20.3 mv (Figure 2B), the negative charge on nanoparticles is due to the presence of functional groups in the amino acids of purified protein, which approves the high constancy of AgNPs. In addition, the FTIR spectra of biological synthesized AgNPs shows the involvement of different functional groups responsible for the stabilization of nanoparticles, which act as a capping or stabilizing agent (Figure 2C). FTIR measurement on AgNPs-mediated by purified protein revealed different absorption peaks at 3435.89, 2079.04, 1633.84, 1384.84, 1219, and 566.95 cm^−1^.In case of AgNPs, a very strong absorption peak shifted toward a lower wave number was observed at 3435.89 cm^−1^, which represented the binding of silver ion (Ag^+^) with hydroxyl and or amine groups in the purified protein. Other bands figured at about 2079.04 cm^−1^, are also remarkable because of the stretching vibration of hydrocarbon (C–H) bonded of alkenes, while the peak at 1633.84 cm^−1^ is also predominant and represents the involvement of amide-I bond (–C=O) of purified protein as a stabilizing agent of AgNPs. Finally, the peak at 1384.84 cm^−1^ represented the C–H bending and peak in 566.95 cm^−1^ could be related to the aromatic ring in the wheat extract.

### 3.2. Exogenously Supplied-Silver Nanoparticle Increased Seed Germination Under Salt Stress

The application of NaCl reduced the average number of seed germination, germination rate, and germination index during the emerging stage in the wheat plant. These attributes were reduced during the emerging stage in comparison to their respective controls. The supplementation of AgNP to salt-stressed plants increased the average number of seed germination, germination rate, and germination index during the emerging stage in comparison to salt-treated plants (Table 1).

### 3.3. Exogenously Sourced-Sliver Nanoparticles Reduces Oxidative Stress Under Salt Stress

**Figure 3:**
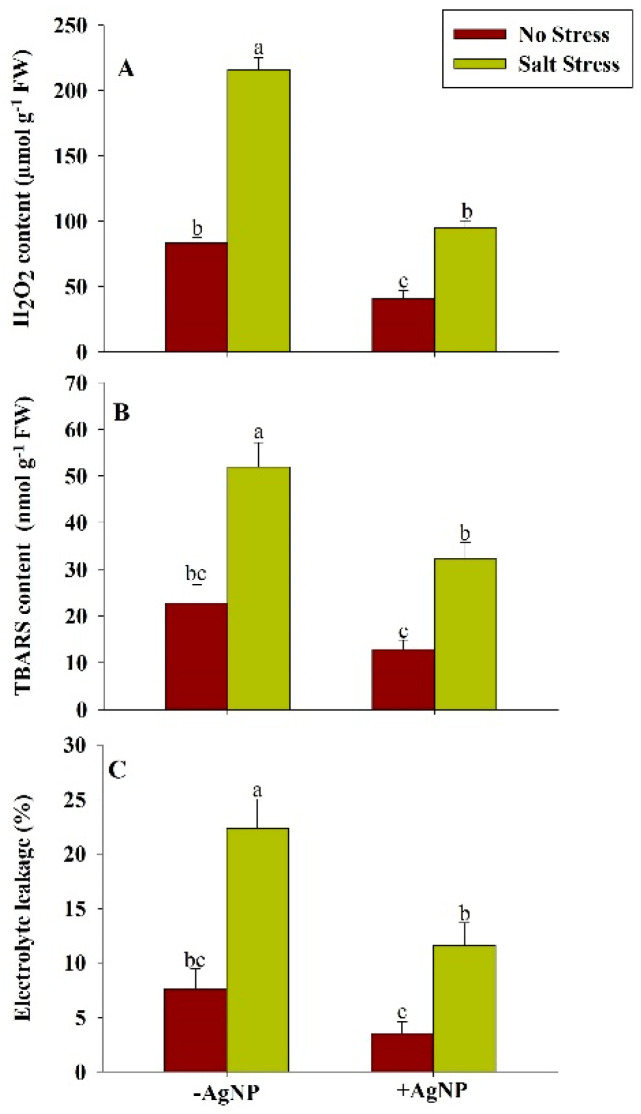
Estimation of oxidative-stress biomarkers: (**A**) Hydrogen peroxide (H_2_O_2_), (**B**) thiobarbituric acid reactive substances (TBARS), and (**C**) electrolyte leakage (EL) content in wheat at 30 DAS. Plants were grown individually with 0 (control), 100 mM NaCl, 300 ppm AgNPs and NaCl combined with AgNPs. Data are presented as treatments mean ± SE (n = 3). Data followed by same letter are not significantly different by LSD test at (*p* ˂ 0.05).

The salt-stress mediated enhanced level of H_2_O_2_ and TBARS in plants were found to be reduced by both individual supplementation of AgNP as well as by its combined supplementation with NaCl. However, the combined supplementation of AgNP + NaCl reduced the H_2_O_2_ content by 56% and TBARS content by 38% compared to their respective NaCl treated plants. The enhanced electrolyte leakages (EL) in wheat plants under salt stress were found about 193% in comparison to the control plant. However, the combined application of AgNP + NaCl inhibits the electrolyte leakage by about 48% in comparison to the NaCl treated plants (Figure 3).

### 3.4. Exogenously Sourced-Silver Nanoparticles Enhanced Antioxidants Under Salt Stress

In response to the salt stress, the increased activity of SOD, APX, GR, and GPX in wheat plants was found to be increased up to 95%, 52%, 84%, and 54%, respectively, in comparison to their respective control plants. However, in response to the separate supplementation of AgNPs or its combined supplementation with NaCl, further increased in the activity of the above-mentioned enzymes has been observed. About 44%, 82%, 89%, and 20% increased activity of SOD, APX, GR, and GPX, respectively, was found by the combined supplementation of AgNPs + NaCl in comparison to their respective NaCl treated plants (Table 2). Salt stress increased the content of GSH by about 14% and reduced the AsA content by about 20% in wheat plants in comparison to their respective control plants. However, the combined application of AgNPs + NaCl further increased the content of GSH up to 18% and enhanced the content of AsA up to 26% in comparison to their respective NaCl treated plants (Table 2).

### 3.5. Exogenously Sourced-Sliver Nanoparticle Maintains Na^+^, K^+^, and Cl^−^ Content Under Salt Stress

The application of NaCl increased the content of Na^+^ and Cl^−^ in both roots and leaf of the wheat plant. These attributes were increased by about 72% and 61% in roots and 75% and 74% in the leaf, respectively, in comparison to their respective controls. However, the supplementation of silver nanoparticles (AgNP) to salt-stressed plants reduced the root Na^+^, Cl^−^ content up to 70%, 73%, respectively, and leafNa^+^, Cl^−^ content up to 80%, 83%, respectively, in comparison to their respective NaCl treated plants (Table 3).In salt-stressed plants, the K^+^ content in root and leaf were decreased by 16% and 26%, respectively, as compared to the control. These attributes were enhanced under cumulative treatments of AgNPs +Salt by 67% and 40%, respectively, in comparison to salt-stressed plants.

### 3.6. Exogenously Sourced-Silver Nanoparticles Modulate Proline Metabolism Under Salt Stress

**Figure 4:**
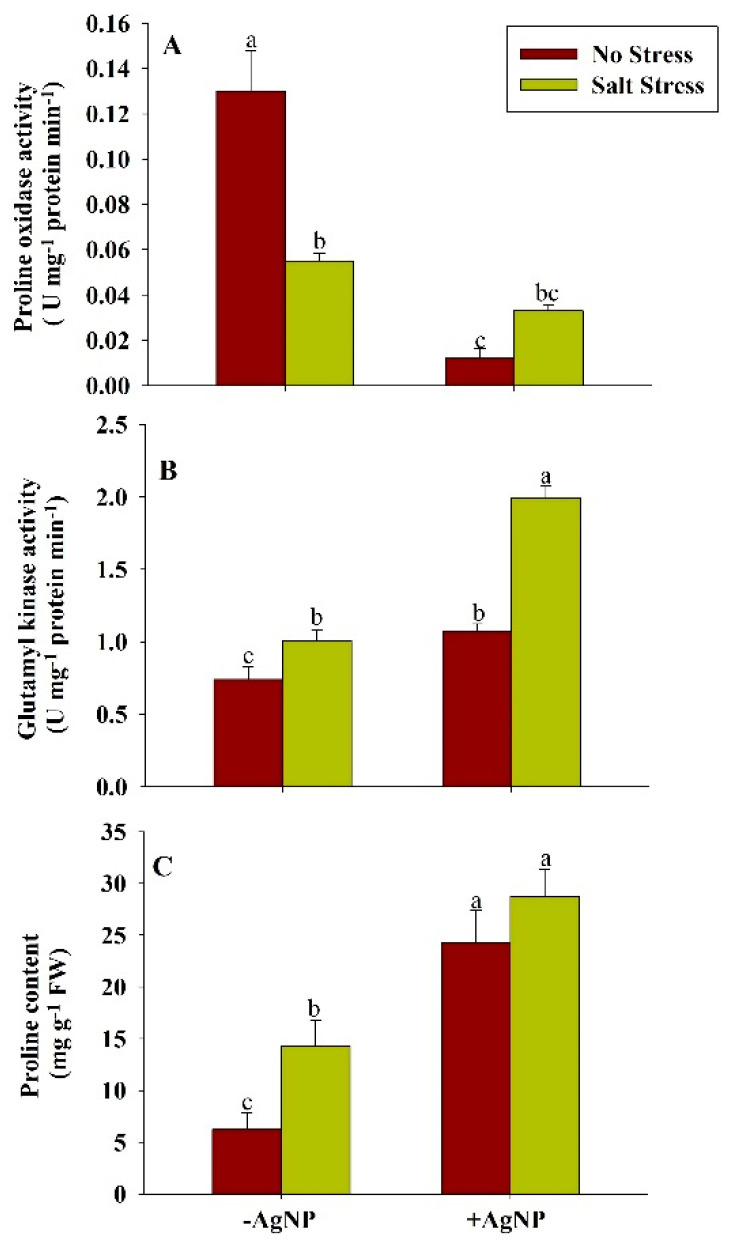
Estimation of proline metabolism: (**A**) proline oxidase activity (**B**) glutamyl kinase activity, and (**C**) proline content in wheat at 30 DAS. Plants were grown individually with 0 (control), 100 mM NaCl, 300 ppm AgNPs and NaCl combined with AgNPs. Data are presented as treatments mean ± SE (n = 3). Data followed by the same letter are not significantly different by LSD test at (*p* ˂ 0.05).

The application of salt stress reduced the activity of proline oxidase (POX) and enhanced the activity of glutamyl kinase (GK) and content of proline in wheat plants in comparison to their respective control plants. Compared to the control plants, about 58% reduced activity of POX and about 36%, 128% increased activity of GK and proline content, respectively, were found in wheat plants grown under salt stress. In response to the individual supplementation of AgNPs and its combined application with NaCl, the activity of POX was reduced and the activity of GK and proline content in wheat plants was enhanced in comparison to their respective control and NaCl treated plants. Compared to the NaCl treated plants, about 40% reduced activity of POX and about 98% and 101% increased activity of GK and proline content, respectively, were found in response to the combined application of AgNPs + NaCl (Figure 4).

### 3.7. Exogenously Sourced-Silver Nanoparticles Enhanced Nitrogen Assimilation Under Salt Stress

**Figure 5:**
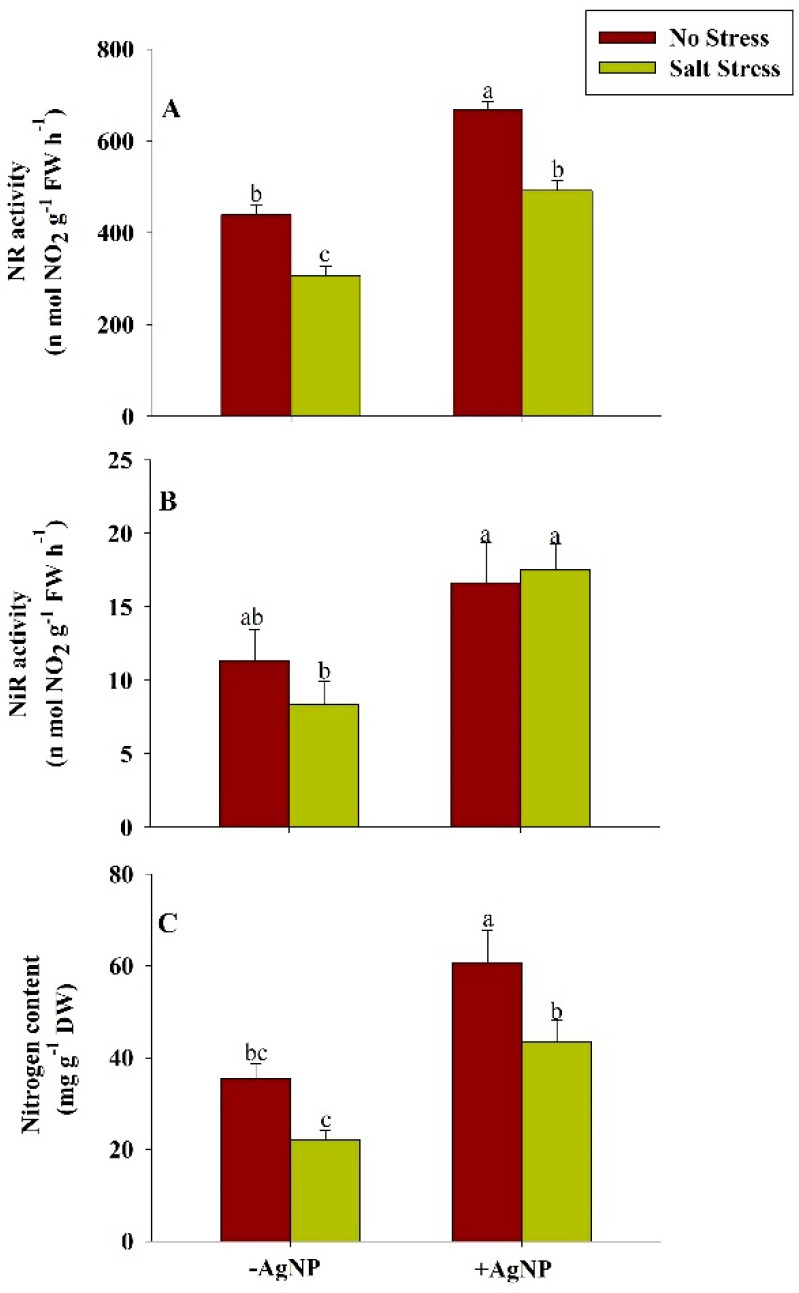
Estimation of nitrogen assimilation: (**A**) nitrate reductase activity (NR), (**B**) nitrite reductase activity (NiR) and (**C**) nitrogen content in wheat at 30 DAS. Plants were grown individually with 0 (control), 100 mM NaCl, 300 ppm AgNPs and NaCl combined with AgNPs. Data are presented as treatments mean ± SE (n = 3). Data followed by the same letter are not significantly different by LSD test at (*p* ˂ 0.05).

In response to salt stress, the activity of NR, NiR and content of N was reduced. These attributes were reduced about 30%, 26%, and 38%, respectively, in comparison to their respective controls. However, the individual supplementation of AgNPs and its combined supplementation with NaCl increased the activity of NR, NiR, and content of N. Compared to the NaCl treated plants, about 60%, 109%, and 98% activities of NR, NiR and content of N respectively were found increased in wheat in response to the combined application of AgNPs + NaCl (Figure 5).

### 3.8. Silver Nanoparticle Application Increased Chlorophyll and Plant Dry Mass Under Salt Stress

**Figure 6:**
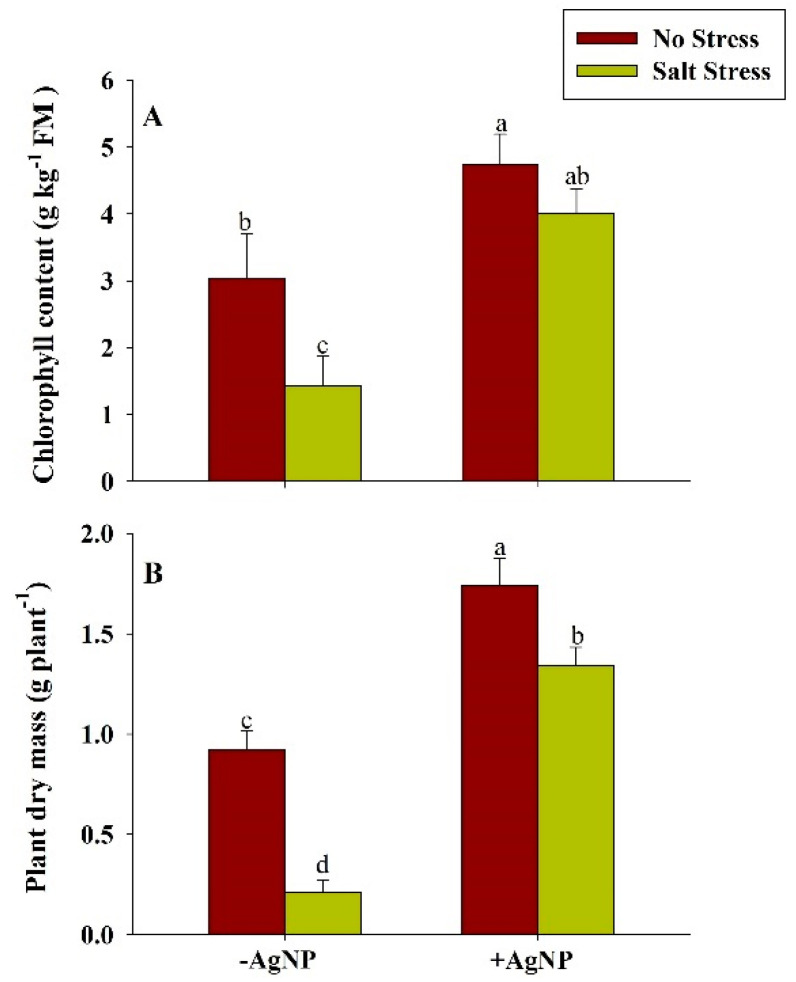
Estimation of (**A**) Chlorophyll content and (**B**) plant dry mass content in wheat at 30 DAS. Plants were grown individually with 0 (control), 100 mM NaCl, 300 ppm AgNPs and NaCl combined with AgNPs. Data are presented as treatments mean ± SE (n = 3). Data followed by the same letter are not significantly different by LSD test at (*p* ˂ 0.05).

Under salt stress, the reduced contents of chlorophyll, and plant dry mass were found in wheat plants in comparison to their respective controls. The application of salt stress reduced the chlorophyll content by about 53% and PDM by about 77% in comparison to their respective control plants. Compared to their respective NaCl treated plants, these attributes were found to be increased in response to the combined application of AgNP + NaCl (Figure 6).

### 3.9. Effect of Silver Nanoparticle Application on Stomatal Distribution on the Leaf Epidermis and ABA Content

**Figure 7:**
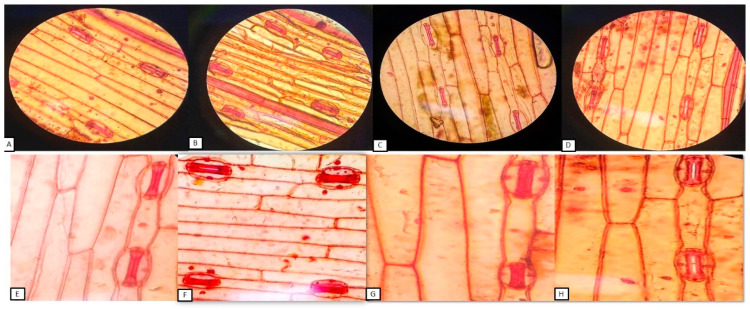
Stomatal area (SA) (**A**–**D**) and stomatal shape coefficient (SSC) (**E**–**H**) at 40× magnification in wheat at 30 DAS. Plants were grown individually with 0 (control), 100 mM NaCl, 300 ppm AgNPs and NaCl combined with AgNPs. (**A**,**E**) = control; (**B**,**F**) = AgNPs; (**C**,**G**) = salt stress; (**D**,**H**) = AgNPs + salt stress.

In salt-stressed plants, SF and SI values significantly decreased by 32%, and 30%, respectively, as compared to the control. AgNPs exposed plants were found to show a remarkable increase in these attributes by 42% and 31%, respectively, than the control. When plants were given foliar application of AgNPs under salinity stress, both of these parameters were observed to increase by 62% and 63%, respectively, than the salt-stressed plants. In AgNPs and AgNPs + NaCl treated plants, SA was found to increase by 8.63% and 11.93% than the control and NaCl exposed plants, respectively. Whereas, the decrease in SSC values were reported in these treatments by 26.19% and 15.72% as compared to the control and NaCl-treated plants. (Figure 7).

**Figure 8:**
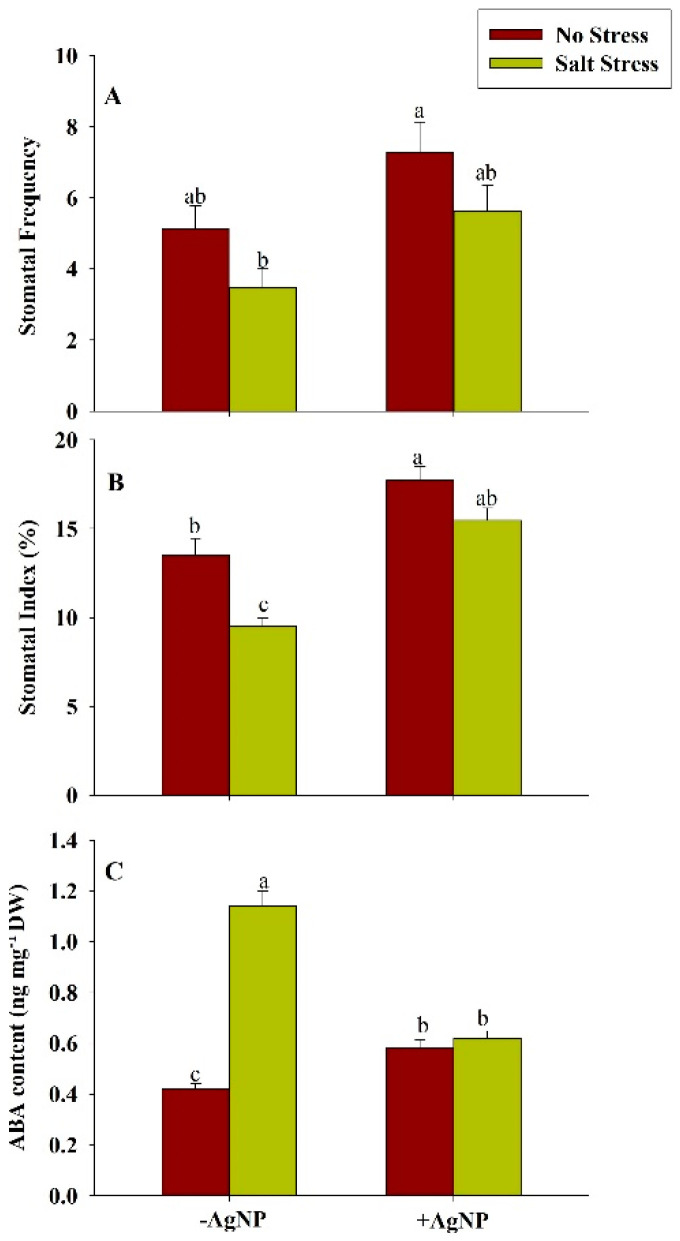
Estimation of stomatal traits: (**A**) Stomatal frequency, (**B**) stomatal index, and (**C**) ABA content in wheat at 30 DAS. Plants were grown individually with 0 (control), 100 mM NaCl, 300 ppm AgNPs and NaCl combined with AgNPs. Data are presented as treatments mean ± SE (n = 3). Data followed by the same letter are not significantly different by LSD test at (*p* ˂ 0.05).

In response to the salt stress, the ABA level was found increased by about 171% in wheat plants in comparison to the control plant. Though, the combined supplementation of AgNP + NaCl reduced the level of ABA by about 46% in comparison to the NaCl treated plants (Figure 8).

## 4. Discussion

Wheat is a staple food and consumed by more than 36% of the world population as the protein and carbohydrate sourced globally. However, the farming of wheat is challenged by salinity stress which significantly induces lower germination and higher accumulation of ions (Na^+^ and Cl^−^) to intrude the metabolic processes of plants. Considering the adverse effects of salinity stress to wheat plants, plant scientists are developing salt-tolerant strategies in plants using different approaches including exogenous protectants in alleviating salt-induced oxidative stress in plants. In the present study, green-synthesized AgNPs from the wheat extract protein have shown their efficacious role in mitigating the stress-inherent traits of wheat under salinity stress. Utilizing the ideology of a greener approach for meeting the sustainable goals for crop improvement and increasing the sovereignty of approaches for food security in the broad-spectrum of the scenario, a successful attempt has been made in modulating the traits responsible for stress-induced mitigation strategies from salinity cues in a response to AgNPs.

The significant roles of AgNPs in mediating plant responses to salinity were observed via a set of parametric analysis that was initiated by analyzing germination parameters for deciphering the involvement of AgNPs in salinity stress responses. Salinity negatively regulated germination parameters such as average seed germination, seed germination rate and index. However, application of AgNPs to the stressed plants reversed these negative effects and ameliorated germination parameters to an appreciable amount during salinity stress. This may happen due to better penetrability of AgNPs into the seed pores [46]; which increases the water uptake efficiency, resulting in coleoptiles elongation, proper seedlings establishment; resulting in a notable acceleration in the germination rate of seedlings of wheat. Furthermore, the enhanced germination percentage has also accelerated the vigorous growth of stems, fresh weight, and root length at the germination stage [47]. The seed primed with AgNPs showed reduced Na^+^ ions translocation from roots to shoots; resulting in increased biomass, photosynthetic pigments, and endogenous proline concentration but decreased in H_2_O_2_ and MDA contents under salt-stressed conditions [48].The results are in agreement with [49] and [50]. In addition to the germination parameters, other criteria including elevation in ROS levels, lipid peroxidation, and stress-induced injuries were analyzed by detecting the hallmark traits such as H_2_O_2_, TBARS, and EL, respectively. An effective deterioration in these stressful attributes under the influence of AgNPs was clearly demarcated in the results for AgNPs supplemented wheat during salinity which was in contradiction from NaCl treated plants. This may result due to the activation of the scavenging systems in the modulation of the defense system via ascorbate-glutathione cycle-mediated [51], thereby inhibiting the lipid peroxidation and formation of their byproducts (TBARS), modulating the K^+^ efflux via inactivation of hydroxyl radical-activated K^+^ outwardly rectifying channels [52].

Biogenic nanoparticles have shown to influence antioxidant responses mediating oxidative stress and plant growth [53]. In our experiment, the biochemical-stress markers such as enzymatic and non-enzymatic antioxidants escalated with high intensity in response to salt stress under NaCl. Enzymatic antioxidants such as SOD, APX, GR, and GPX act as the first line of defense in stress-induced responses, while the non-enzymatic antioxidant entities such as AsA and GSH are mainly regarded as the buffer-system of the plant cells [54]. Since these parameters are exclusively acquired during stressful cues; an increase in antioxidant content under NaCl and a significant reduction in response of AgNPs satisfy the considerate physiological responses with accordance of the given treatment by maintaining the homeostatic equilibrium between cellular moieties.

Salinity tolerance is a complex trait, which involves plant-specific traits including physiological mechanisms such as osmotic tolerance, exclusion of toxic ions, and tissue tolerance. Osmotic tolerance involves all adjustments in plants by the production of osmoprotectants such as proline, uptake of K^+^ and translocation of K^+^ in shoots, exclusion of the toxic amount of Na^+^ in roots and shoots [55]. The ability to maintain K^+^ ion during salinity stress is a requirement of salt-tolerant plants [56]. In this study, it is shown that K^+^ levels decreased in salt-stressed plants, despite the higher accumulation of Na^+^. Our findings revealed that the uptake of crucial K^+^ ions is antagonistic to excessive Na^+^ and Cl^−^ ions in wheat during salinity. In the present study, AgNPs treatment resulted in the reduced level of both Na^+^ and Cl^−^ and increased level of K^+^ ion in leaf as well as in roots. This happens perhaps due to the high K^+^/Na^+^ ratio in plant tissues, reduction in Na^+^-induced K^+^ efflux from root and leaf resulting in high intracellular K^+^-retention or reduction in K^+^-efflux [57].

Furthermore, to determine the adaptive strategies adopted by plants under stress conditions, we analyzed proline concentration and its metabolic pathways during salinity stress. Proline concentration significantly increased under NaCl treatment corresponding to glutamic acid activation, and inhibition of proline oxidation via the action of POX to other soluble compounds such as Pyrroline-5-carboxylate (P5C) [58]; and over-expression of GK initiating a cascade pathway of reactions; resulting in biosynthesis of proline from P5C substrate via enzymatic action of P5C reductase. Proline actively participates in abiotic stress responses and show multifaceted roles such as a coordinating role with the AsA-GSH cycle-mediated protection of cellular membranes against the ROS-assisted oxidative stress [59]. Proline also adjusts osmotic balances of cells, acts as a source of energy for the recovering tissues, alleviates the cytosolic acids concentration, mediates apoptotic pathways in response to ROS-accumulation, results in a cell-cycle arrest or autophagy. Therefore, the AgNPs-mediated enhanced proline content along with up-regulation in the activity of GK, and repressed activity of POX justify the outcomes of the study. For encouraging the eco-sustainable cropping methodology, nitrogen is regarded as a fundamental nutrient due to its wide array functions such as regulating enzymatic activities, photosynthesis, protein synthesis, metabolism of antioxidants, and osmolytes [60].

In the present study, nitrogen content significantly enhanced along with its key-regulatory enzymes (NR and NiR), suggesting an innovative approach for decreasing the reliability of chemical fertilizers especially on the extrinsic supply of nitrogen [61]. The decrease in enzymatic activity of NR and NiR under salinity may be due to the interferes with the nitrogen acquisition and utilization and influencing uptake efficiency of nitrate ions which is further inhibited by Cl^−^ ions or due to changes in the integrity of plasmalemma for the membrane proteins [62]. However, our findings revealed that the foliar application of AgNPs mitigates these adversities caused by salinity in the stressed plants, suggesting that AgNPs ceased the inhibitory or antagonistic effects of Na^+^ and Cl^−^ on efficacy of nitrogen uptake and assimilation. Additionally, nitrogen is an evidential parameter that is directly correlated with the chlorophyll content [63]. Furthermore, our findings demonstrated the positive interdependence of chlorophyll content and accumulation of dry mass under AgNPs, which has been proven to be an efficient method for improvising the accuracy of management strategies. The increase chlorophyll content also indicates AgNPs-mediated enhanced PS-II quantum efficiency [64] enhanced fluorescence quenching efficiency [65]; the increased activity of RuBisCO, and PEP carboxylase [66]. Additionally, the effects of AgNPs on stomatal index, stomatal frequency, stomatal area, SSC and ABA during salinity stress were also analyzed. The permissible change in stomatal dynamical traits helps in modification of turgor pressure in guard cells, aperture acclimatization, and optimization of the rate of gaseous exchange in response of both internal and external environmental cues [67]. The value of SSC was observed to increase under salinity stress and a more circular shape of the stomatal surface was observed. The circular shape of the stomatal area is beneficial for mitigating or alleviating stress conditions [68]. These adaptations also facilitate enhancement in water use efficiency and augmentation of plant productivity [69]. Under salinity conditions, it is reported that ABA alters the physiological responses such as promoting seed dormancy; stomatal closure; leaf senescence; embryonic morphogenesis; and accumulation of proteins, and lipids to imitate the stress [70]. Xu et al. [71] reported that leaf ABA significantly increased under salinity stress and highly correlated with leaf Na^+^ concentration. They suggested that under salinity stress, leaf ABA may participate in the regulation of leaf growth, and leaf Na^+^ would be at least partly responsible for increased ABA levels in bean plants. Since accumulation of K^+^ ions increase [72], ABA content decreases in the response to AgNPs under salinity stress. All the traits studied in the current research provide insight into the view of applicability and positive aspects of AgNPs in modifying traditional agricultural practices in collaboration with nanosciences.

## 5. Conclusions

This study emphasizes the prodigious interlinking between sustainable agricultural practices and nanosciences where green synthesized AgNPs from wheat remarkably aided in ameliorating the adverse effects of salinity stress on various plant responses. The AgNPs successively reduced the adversities of salt-stress conditions in wheat and positively regulated parameters analyzed such as seed germination traits, K+ ionic concentration, ABA production, antioxidant machinery, proline accumulation, nitrogen content and its regulatory enzymes (NR, NiR), chlorophyll content and stomatal traits. The efficacy of AgNPs in enhancing plants’ survival under excess NaCl conditions and their role in improvising nitrogen-related traits are the efforts made to reduce the dependency on external supplementation of chemical fertilizers for crop improvement. However, fingerprinting of various stress-mediated traits or responses is further needed to be taken into consideration in different species to correlate the plant-specific interaction. In addition, risk assessment NPs are needed to evaluate for the real-time tracing of comparative genome-induced responses under salinity stress. With an utmost necessity to identify novel diagnostic and prognostic biomarkers for predicting the effects of compounds induced by AgNPs in plants, the upcoming years in the research field will definitely come up with the spatial mapping of transcripts induced under various stresses in retaliation with green-synthesized AgNPs. Further studies are needed for a profound understanding of traits influencing gene interactions of plants in the response to green-synthesized nanoparticles.

## Figures and Tables

**Table 1:** Average number of seeds germination, germination rate (%), and germination index (%) in wheat (*Triticum aestivium*) seedlings exposed to 100 mM salt stress and supplemented with silver nanoparticle (300 ppm; AgNP) at 20 days after sowing (DAS). Data followed by the same letter are not significantly different by LSD test at (*p* < 0.05).

Treatments	Seeds Germinated	Germination Rate (%)	Germination Index (%)
Control	17.67 ± 1.31 b	73.62 ± 3.16 b	0.0 ± 0.0 c
NaCl	8.33 ± 1.29 c	68.04 ± 2.47 c	92.4 ± 3.24 b
AgNP	29.33 ± 3.93 a	84.7 ± 4.22 a	115.1 ± 7.83 a
AgNP + NaCl	18.33 ± 1.69 b	76.37 ± 3.37 ab	103.7 ± 4.16 ab

**Table 2:** Superoxide dismutase activity (SOD; U mg^−1^ protein min^−1^), ascorbate peroxidase activity (APX; U mg^−1^ protein min^−1^), glutathione reductase activity (GR; U mg^−1^ protein min^−1^), glutathione peroxidase activity (U mg^−1^ protein min^−1^), reduced glutathione content (GSH; nmol g^−1^ fresh mass), and ascorbate content (AsA; mg g^−1^ FW) in wheat (*Triticum aestivium*) exposed to salt stress (100 mM) and supplemented with silver nanoparticles (AgNPs; 300 ppm) at 30 days after sowing (DAS). Data fallowed by the same letter are not significantly different by LSD test at (*p* ˂ 0.05).

Antioxidants	Control	NaCl	AgNP	AgNP + NaCl
SOD	30.33 ± 4.97 b	59.14 ±6.13 a	22.14 ± 4.54 b	33.12 ± 6.72 b
APX	3.2 ± 0.73 c	4.86 ± 0.93 b	7.7 ± 0.54 a	8.86 ± 0.66 a
GR	3.5 ± 0.62 c	6.44 ± 0.53 b	10.98 ± 1.68 a	12.19 ± 1.65 a
GPX	0.026 ± 0.002 d	0.04 ± 0.002 c	0.066 ± 0.001 a	0.048 ± 0.001 b
GSH	320 ± 12.06 c	365 ± 16.08 bc	413 ± 19.62 ab	431 ± 23.77 a
AsA	0.92 ± 0.027 b	0.74 ± 0.056 c	1.21 ± 0.027 a	0.93 ± 0.072 b

**Table 3:** Root and leaf Na^+^, Cl^−^ (mg g^−1^ DW) content, root and leaf K^+^ content (mM) in wheat (*Triticum aestivium*) exposed to salt stress (100 mM NaCl) and supplemented with silver nanoparticles (AgNPs; 300 ppm) at 30 days after sowing. Data fallowed by the same letter within the column are not significantly different by LSD test at (*p* ˂ 0.05).

Treatments	Root			Leaf		
	Na^+^ Content	Cl^−^ Content	K^+^ Content	Na^+^ Content	Cl^−^ Content	Leaf K^+^
Control	13.4 ± 1.75 b	15.6 ± 1.94 b	246 ± 9.44 c	11.34 ± 1.57 b	10.12 ± 1.55 b	226 ± 9.49 b
NaCl	23.03 ± 2.29 a	25.06 ± 2.64 a	207 ± 8.18 d	19.85 ± 3.48 a	17.56 ± 2.75 a	167 ± 11.65 c
AgNP	6.7 ± 1.01 c	4.21 ± 1.11 d	401 ± 10.71 a	2.72 ± 1.10 d	2.13 ± 0.57 c	346 ± 9.59 a
AgNP +NaCl	6.85 ± 1.84 c	6.74 ± 1.63 c	345 ± 8.56	4.01 ± 1.38 c	2.98 ± 0.72 c	234 ± 12.04 b

## Supplementary Materials

The following are available online at https://www.mdpi.com/2218-273X/10/11/1506/s1.
