# Supplementary material for: Silver Nanoparticle Regulates Salt Tolerance in Wheat Through Changes in ABA Concentration, Ion Homeostasis, and Defense Systems
Source: Biomolecules. 2020 Nov 2;10(11):1506. doi: 10.3390/biom10111506 (PMC7694077; doi:10.3390/biom10111506)
Supplement: Supplementary file 1 [file biomolecules-10-01506-s001.zip › biomolecules-969257-supplementary.docx]

**3.1 Estimation of oxidative stress**

The leaf lipid peroxidation was determined via the estimation of thiobarbituric acid reactive substances (TBARS) contents by following the method of Dhindsa et al. (1981). According to this method about 500 mg of fresh leaf tissue were crushed in 0.25% 2-thiobarbituric acid (TBA) in 10% trichloroacetic acid (TCA). After that the aliquot were heated at 95^⸰^C for about 30 minutes. Following the quick cooling, the mixture was centrifuged at 10,000 ⅹ g for about 10 min. Take 1mL supernatant and add it 4 mL 20% TCA containing 0.5% TBA. Take the absorbance of the supernatant at 532nm and nullify the non-specific turbidity via subtracting the absorbance of the same at 600nm. The calculation of TBARS content was done by using the extinction coefficient (155 mM^-1^ cm^-1^).

The determination of H_2_O_2_ content was done by following the method of Okuda et al. (1991), in which about 200mg leaf sample were crushed in 200mM ice cold perchloric acid. By following the centrifugation (1, 200 ⅹ g for 10 min.) the supernatant of perchloric acid was neutralized by adding 4M KOH. After that, centrifuge the aliquot at 500 ⅹ g for 3 min. In a final volume of 1.5 mL, 1mL of the eluate, 400 mL of 12.5 mM 3-(dimethylamino) benzoic acid (DMAB) in 0.375Mphosphate buffer (pH 6.5), 20 mL of peroxidase (0.25 unit), and 80 mL of 3-methyl-2- benzothiazolinehydrazone (MBTH) were added . After that peroxidase was added at 25 ^⸰^C and the absorbance was recorded at 590nm.

The electrolyte leakage were measured by thoroughly washed samples with sterile water followed with weighing and immersed the samples in closed vials containing 10mL deionized water. After that, the samples were incubated at 25 ^◦^C for 6 h using shaker and electrical conductivity (EC) was determined (C_1_). After that the samples were placed at 90^◦^C for 2 h and EC was monitored after achieving the equilibrium at 25 ◦C (C_2_).

**3.2 Estimation of ion content:**

The estimation of Na^+^ and Cl^-^ content was determined via the digestion of plant samples using Tri acid mixture (TAM): a sulfuric acid, perchloric acid and nitric acid mixture in the ratio of 5: 4: 10. By using flame photometer the estimation of Na^+^ content was determined; however the determination of Cl^-^ content was done by titration against 0.02 N silver nitrate solution using 5% K_2_CrO_4_ as indicator.

The estimation of K^+^ content was determined by immediate frozen of leaf sample in liquid nitrogen. For determination, the defrost leaf sample were set in to 1.5 ml microcentrifuge tubes having basal opening, through which cell sap allows but not the fragments of tissue. The samples were centrifuged at 11 600 ⅹ g for 3 min. in a microcentrifuge. The collected samples were taken for determination of K^+^ content by using flame photometer. The analysis of K^+^ content was done by following the method of Walker et al., 1995.

**3.3 Estimation of enzymatic and non-enzymatic antioxidants**

About 200 mg leaf samples were crushed in 0.05% (v/v) Triton X-100 and 1% (w/v) PVP in potassium-phosphate buffer (100 mM, pH 7.0) containing extraction buffer. The aliquot was spun 15,000 ×g for 20 min at 4 ◦C in centrifuge and the obtained supernatant was used for the assay of enzymes superoxide dismutase (SOD; EC 1.15. 1.1), ascorbate peroxidase (APX; EC 1.11.1.11), glutathione reductase (GR; EC 1.6.4.2), glutathione peroxides (GPX; EC 1.11.1.9).

The activity of SOD was determined according to the method of Beyer and Fridovich (1987) and Giannopolitis and Ries (1977). The determination of SOD was done by monitoring the 50% inhibition of photochemical reduction of NBT. The reaction mixture of about 5.0 ml containing 5.0 mM HEPES (pH 7.6), 50 mM Na_2_CO_3_ (pH 10.0), 63 μmol NBT, 0.1 mM EDTA, 13 mM methionine, 0.025% (v/v) Triton X-100, 1.3 μmol riboflavin. The enzyme extract was illuminated for 15 min (360 μmol m–2s–1) and the control set was not illuminated to correct for background absorbance. The amount of enzyme that inhibited the NBT reduction by 50% at 560 nm is defined as the one unit of SOD.

The activity of APX was determined according to the method of Nakano and Asada (1981). APX activity was determined by the decrease in absorbance of AsA at 290 nm due to its enzymatic breakdown. The reaction mixture of about 1.0 ml contained 50 mM phosphate buffer (pH 7.0), 0.5 mM ascorbate, 0.1 mM H_2_O_2_, 0.1 mM EDTA and the enzyme extract. The activity of APX was calculated by using the extinction coefficient 2.8 mM^–1^cm^–1^. The amount necessary to decompose 1.0 μmol of substrate per min at 25^o^C is defined as the one unit of APX.

The determination of GR activity was done by following the method of Foyer and Halliwell (1976) by observing the GSH-assisted NADPH oxidation. The assay mixture contained 0.5 mM oxidized GSH, phosphate buffer (25 mM,pH 7.8), the enzyme extract and 0.2 mM NADPH. The activity of GR was estimated via using extinction coefficient at 6.2 mM^−1^ cm^−1^. One Unit of the enzyme was amount necessary to decompose 1µmol of NADPH min^−1^ at 25 ^◦^C.

The determination of GPX activity was done by following the method of Hasanuzzaman et al. (2012), in which 1mL of reaction mixture containing 50 mM potassium phosphate buffer with pH 7, 1 mM NaN_3_, 1 mM EDTA, 1 U mL^−1^ GR, 0.2 mM NADPH, 0.25 mM H_2_O_2_ and 1 mM GSH. The NADPH oxidation was spectrophotometrically recorded at 340nm and the enzyme activity was estimated via using extinction coefficient at 6.2 mM^−1^ cm^−1^. One Unit of the enzyme was amount necessary to decompose 1µmol of NADPH min^−1^ at 25 ^◦^C.

The content of reduced glutathione (GSH) was determined via enzyme recycling procedure offered by Griffith (1980). According to this method about 500mg of leaf sample were crushed in 2.0 ml of 5% sulfosalicylic acid (SSA). The aliquot was spun at 10,000*×g* for 10 min in centrifuge. After that 0.6 mL phosphate buffer and 40 μl DTNB were added to 0.5mL of supernatant. After two min, the absorbance was recorded at 412 nm.

The content of AsA was estimated via the method of Smith et al. (1985) which was adopted by Wei et al. (2015).

**3.4. Estimation of proline oxidase, glutamyl kinase activity and proline content:**

The proline oxidase activity was determined via the method of Huang and Cavalieri(1979). About 500mg fresh leaf samples were homogenized in Tris–HCl buffer (0.1 M; pH 7.5) at temperature 4 °C. After the centrifugation of aliquot at 30,000×*g* for 30 min, the pellets were collected and were dissolved in 1.0 ml tricine and KOH buffer (pH 7.5) with 6.0 M sucrose containing mixture. The extract collected was used for the assay of enzyme. The assay mixture contained 1.2 ml of 50 mMTris–HCl buffer (pH 8.5), 0.1 ml of 0.5 mM NADPH, 1.2 ml of 5.0 mM MgCl_2_, 0.1 ml of 0.5 mM NADPH, 0.1 ml of 1.0 mM KCN, 0.1 ml of 0.1 M proline, 0.1 ml of 1.0 mMphenazinemethosulfate, in a final volume of 3.0 ml, and 0.1 ml of 0.06 mM 2,6 dichlorophenol indophenols (DCPIP). The initiated point reaction occurred by the addition of proline and the absorbance was spectrophotometrically recorded at 600 nm at 25 °C. The activity of proline oxidase was expressed in U mg^−1^ protein. One unit (U) of enzyme activity is defined as mM DCPIP reduced min^− 1^ mg^−1^ protein.

The glutamyl kinase activity was determined via the method given by Hayzer and Leisinger (1980) having slight modification with procedure of proline oxidase activity. This method was adopted by Khan et al. (2014).

The content of proline was estimatedspectrophotometrically by the method given by Bates et al. (1973). According to this method about 300mg of leaf sample were crushed in 3 mL of 3% sulphosalicylic acid. The homogenate collected were reacted with 1mL acid ninhydrin and 1mL glacial acetic acid for about 1h in test tubes, left in a water bath at temperature 100 ^◦^C. The toluene was added to the aliquot for the extraction of mixture and the absorbance was recorded at 520nm using l-proline as a standard.

**3.5 Estimation of NR, NiR activity and N content**

The estimation of leaf NR activity was determined by following the method given by Kuo et al. (1982). According to the procedure about 1.0g of fresh leaf were crushed by using mortar and pestle and then stored at -80 ºC. After thawed the powder for 10 min. at 4°C, the sample were homogenized in 250 mMTris-HCl buffer (pH 8.5) containing 1.0 mM EDTA, 10 mM cysteine, 1.0 mM DTT, 20 μM FAD and 10% (v/v) glycerol. The aliquot were spun at 10,000*×g* in centrifuge at 4ºC for 30 min. The assay mixture containing 0.065 M HEPES (pH 7.0), 10 mM KNO_3_, 0.5 mM NADH in 0.04 mM phosphate buffer (pH 7.2) and final volume of enzyme of about 1.5mL. The addition of NADH initiates the starting of reaction. Within 15min, the reactionswere terminated by adding 1.0 ml of 1.0 N HCl solution containing 1% sulphanilamide. After the adding 1.0 ml of 0.02% aqueous N-1-napthylethylene-di–amine-dihydrochloride (NED), the absorbance was recorded at 540nm after 10min of incubation.

The NiR activity was determined via a method given by Ramarao et al. (1983) which was adopted by Hussain et al. (2020).

The N content in leaf was estimated in acid-peroxide digested material using the method of Lindner (1944). About 10 ml Aliquot from digested material was taken in a volumetric flask. To neutralize the excess acid and prevent turbidity, 2.0 ml of sodium hydroxide (2.5 N) and 1.0 ml of 10% sodium silicate solutions were added to volumetric flask. To this, distilled water was added and the volume was made up. In a 10 ml graduated test tube, 5.0 ml from aliquot was taken and added with 0.5ml Nessler’s reagent. The volume was maintained with distilled water. For the development of maximum color, the contents of tubes were allowed to stand for 5min. Absorbance was taken at 525 nm using spectrophotometer. The content of N was estimated using a standard graph prepared by graded dilutions of ammonium sulphate. Photosynthetic nitrogenuse efficiency (NUE) was estimated by the ratio of rate of photosynthesis to N content per unit leaf area.

**3.6 Estimation of chlorophyll content and plant dry mass**

The fully expanded fresh leaf was taken and then cut it in to strips about 2mm in width. The extractions of chlorophyll were done from 25-50mg fresh leaf tissue by DMSO containing 96% ethanol or 80% acetone. In the non-lethal DMSO method, the leaf tissues were immersed in glass tubes at temperature 65^◦^C with regular shaking until the leaf tissue becomes colorless. Record the absorbance spectrophotometrically at 665nm and 648nm. The content of Chla andChl b were estimated by applying the equation given by Barnes et al. 1992.

From the pots the plants were uprooted carefully, washed for removing adhering material and dust and after that following the drying of sample in hot air oven at 80 °C till constant weight and note dry mass weight.

**3.7 Measurement of Stomatal Traits**

The Stomatal Index (SI) (%) was estimated by using the formula illustrated by Xu & Zhou (2008):-

SI (%) = [S÷ (E+S)] ×100where, S= Number of stomata per unit area; E= Number of epidermal cells in the same unit area

Stomatal Frequency (SF) {no. of stomata per mm^2^) was determinedby the formula given by Salisbury (1927):-

SF= [S in entire FOV ÷ Area of FOV]

where, S= Number of stomata per unit area; FOV= Field of View= 0.65 mm^2^ (at 40X)

**3.8. Estimation of ABA content**:

The determination of abscisic acid (ABA) content was done following the method given by Hung and Kao (2003). According to the protocol the fresh leaf samples were frozen immediately in liquid nitrogen and crushed in to fine powder. After following the homogenization of powder in an extraction solution (80% methanol containing 2% glacialaceticacid) the centrifugation of extract has been done and elute via the polyvinyl pyrrolidone column and C18 cartridges to take out the plant pigments and other non-polarcompounds which shows interference in the immune assay. The extracts were concentrated to dryness by using vacuum evaporation and before following enzyme-linked immunosorbent assay (ELISA), the dry sample were resuspended in Tris-buffered saline. The determination of ABA were done via ABA immune assay detection kit (PGR-1;Sigma-Aldrich,St.Louis, MO,USA) as per the procedure found in manual. The values were taken at 405nm and the content of ABA was calculated from a calibration curve by using ABA standard.
